# Analysis of Toxic Element Levels and Health Risks in Different Soybean Species (*Glycine max*, *Vigna radiata*, *Vigna angularis*, *Vigna mungo*)

**DOI:** 10.3390/nu16244290

**Published:** 2024-12-12

**Authors:** Juan R. Jáudenes-Marrero, Greta Giannantonio, Soraya Paz-Montelongo, Arturo Hardisson, Javier Darias-Rosales, Dailos González-Weller, Ángel J. Gutiérrez, Carmen Rubio, Samuel Alejandro-Vega

**Affiliations:** 1 Toxicology Area, University of La Laguna, Tenerife, Canary Islands, 38071 La Laguna, Spain; jjaudene@ull.edu.es (J.R.J.-M.); atorre@ull.edu.es (A.H.); alu0100881288@ull.edu.es (J.D.-R.); dgonzal@ull.edu.es (D.G.-W.); ajguti@ull.edu.es (Á.J.G.); salejand@ull.edu.es (S.A.-V.); 2 School of Pharmaceutical Sciences and Health Products, University of Camerino, 62032 Camerino, Italy; 3 Health Inspection and Laboratory Service, Canary Health Service, S/C de Tenerife, Tenerife, Canary Islands, 38006 Santa Cruz de Tenerife, Spain

**Keywords:** soybean, toxic elements, exposure assessment, risk characterization

## Abstract

**Background:** Soybeans are a widely consumed legume, essential in Western diets and especially prominent in vegan and vegetarian nutrition. However, environmental contamination from anthropogenic sources, such as industrial emissions, wastewater, and pesticide use, has led to the accumulation of non-essential and toxic elements in legumes, potentially impacting human health. **Method:** This study quantified the levels of 11 potential toxic elements (Al, B, Ba, Cd, Co, Cr, Li, Ni, Pb, Sr, V) in 90 samples of four soybean species (*Glycine max*, *Vigna radiata*, *Vigna angularis*, *Vigna mungo*) using inductively coupled plasma optical emission spectrometry (ICP-OES). **Results:** Results showed that boron had the highest mean content (9.52 mg/kg ww), followed by aluminum (6.73 mg/kg ww). Among the toxic metals, cadmium was most concentrated in green soybeans (0.03 mg/kg ww), and black soybeans had the highest level of lead (0.07 mg/kg ww). Based on an average soybean consumption of 50 g/day, no immediate health risk was detected. However, lithium and nickel were present in substantial amounts, with lithium contributing 31.43–48.57% and nickel 6.81–39.56% of their respective provisional daily intake limits, especially from red soybeans (*V. angularis*). **Conclusions:** This study highlights the importance of monitoring toxic elements in soybeans and calls for stricter environmental management practices to minimize contamination, ensuring the safety of soy products as their global consumption rises.

## 1. Introduction

Legumes are a dietary staple for people around the world. However, the environmental pollution caused by human activities like wastewater, industrial emissions, sewage irrigation, burning of fossil fuels, use of chemical pesticides, etc., could cause an increment in the elements content in these crops [1,2]. This could pose a potential toxicological risk from the consumption of legumes, so a risk assessment of their consumption is necessary [3,4,5].

The soybean is a plant belonging to the family Leguminosae, subfamily Papilionoidea, and it originated in Eastern Asia. Soybeans have been grown as a food crop for thousands of years in China and other countries of East and Southeast Asia and constitute to this day an important component of the traditional popular diet in these regions [6,7].

Nowadays, soybeans are widely used in human nutrition, in animal feed, as a fertilizer, and for biodiesel fuel production, and recently, it has also found use in cosmetics and the pharmaceutical field [8,9]. In human nutrition, soybeans are consumed in both non-fermented and fermented forms [10,11,12,13]. Among the non-fermented soy foods, the most famous are soy milk, meat analogs (for example “veggie burgers”), tofu, soy sprouts, and soy protein. Fermented foods, however, include miso, soy sauce, and tofu (fermented tofu).

Since the 1950s, soy production has increased 15-fold and shifted from Asia to the American continent, which accounts for more than 93 percent of the world’s soybean production. Brazil is the largest soybean producer in both the region and the world. According to the USDA (United States Department of Agriculture) data, Brazil produced 160 million tons of soybeans in the 2022/23 season. In global production, the USA (United States of America) follows Brazil with 116 million tons [14]. According to the IGC (International Grains Council) data, the world soybean consumption was 359 million tons in the 2022/23 season. Ranking first with 100.8 million tons in global soybean imports in the 2022/23 season, China is estimated to import 102 million tons in the 2023/24 season. While the imports by the European Union countries will remain around 13 million tons, Mexico will continue to import around 6 million tons [14,15].

While “soybean” seems like a straightforward term, its classification in Chinese popular culture can be misleading. It encompasses four distinct legume species: yellow soybeans (*Glycine max*), green soybeans (*Vigna radiata*), red soybeans (*Vigna angularis*) and black soybeans (*Vigna mungo*). Though all belong to the legume family, they represent separate botanical species. In fact, in the scientific field, only the botanical species *Glycine max* is recognized as a soybean [16]. In particular:

- Yellow soybeans (*Glycine max*): A powerhouse among crops, yellow soybeans thrive in warm regions from the tropics to temperate zones. These annual, upright plants can grow up to 2 m tall and produce pods 3–8 cm long containing 2–4 seeds, typically yellow in color [17].
- Green soybeans or mung beans (*Vigna radiata*): Originally from India but popular across East Asia, the mung bean is a fast-maturing legume suited for warm seasons. As an annual crop often grown in rotation with cereals, it boasts plants around 60–75 cm tall that yield roughly 7.5 cm long pods packed with a dozen or so small, green seeds [17].
- Red soybeans or azuki beans (*Vigna angularis*): The runner-up to yellow soybeans in economic importance across Eastern markets, the Azuki bean thrives in warm temperate and subtropical climates. These upright plants reach 30–60 cm in height and produce pods similar to mung beans. The most common seeds are a deep red color, boasting a size two to three times larger than mung beans [17].
- Black soybeans or mungo beans (*Vigna mungo*): Hailing from central Asia (India), the black mungo bean prefers temperatures between 25–35 °C for optimal growth. These plants can reach heights of 30–100 cm, sporting large hairy leaves and pods 4–6 cm long. Each pod shelters 4–10 black beans [17].

The differential macronutrient profiles among soybean species result in unique technological and sensory attributes. While flavonoids, anthocyanins, and carotenoids significantly influence color [18,19], variations in other macronutrients also contribute to species-specific characteristics. For example, green and black soybeans exhibit a lower carbohydrate content compared to other varieties. Yellow soybeans serve as a primary source for processed products like soy milk and tofu. Green soybeans have a historical application in soybean flour production. Black soybeans, characterized by a mild flavor, are versatile in culinary applications. Red soybeans, with their inherent sweetness, are frequently used in baked goods [20,21].

As said before, considering the ability of crops to absorb and accumulate different elements from soil, water, and the environment, it is necessary to determine the levels of certain elements that could be toxic to human health [22]. Then, the determination of non-essential and toxic elements (Al, B, Ba, Cd, Co, Cr, Li, Ni, Pb, Sr, V) in legumes is interesting. Moreover, European legislation, to which the country where this study was conducted belongs, has established in its legislation limits the presence of some of these potentially toxic elements in the legume group. A parametric value is established for lead of 0.20 mg/kg and for cadmium of 0.04 mg/kg at the Commission Regulation (EU) 2023/915 [23].

Aluminum (Al) is a neurotoxic element that tends to accumulate in the brain, bones, liver, and kidneys. Prolonged exposure to high levels of Al has been related to neurodegenerative diseases such as Alzheimer’s [24,25].

Cadmium (Cd) has toxic effects due to its high half-life and bioaccumulation [26]. It can affect the renal system, causing irreversible damage to the renal tubules, which are involved in nutrient reabsorption mechanisms [27,28,29].

Lead (Pb) is a neurotoxic element that tends to accumulate in the body, causing serious damage to the central nervous system, especially in developing children and fetuses [30]. This can cause kidney disease, gastrointestinal tract disorders, and Alzheimer’s [31].

Boron (B) is an essential element for plant organisms [32] and that is why its concentration in vegetables and legumes is high [33]. Studies on experimental animals have shown that an excessive B intake can lead to intoxication, causing problems in the digestive system, damage to specific kidney cells, and skin peeling [34].

An excessive intake of barium (Ba) can cause tachycardia, hypertension, hypotension, muscle weakness, and paralysis. An excessive intake of cobalt (Co) is unusual; however, it could cause the alteration of calcium homeostasis as it blocks the cellular calcium channels. High chromium (Cr) intake can trigger chronic kidney failure, dermatitis, bronchitis, asthma, etc. [35,36,37].

Excessive intake of lithium (Li) can lead to altered consciousness, tremors, ataxia, apathy, etc. [38,39]. Strontium (Sr) can cause a deficiency of phosphorus and its accumulation in the bones could lead to an increase in bone density [40]. Therefore, gastrointestinal disorders have been observed in humans derived from excessive vanadium (V) intake [35]. Nickel (Ni) is an essential element for plants [41] and individuals with Ni hypersensitivity or kidney problems are susceptible to harm from Ni ingestion [42].

The objectives of this study were (i) to determine the content of non-essential (B, Ba, Co, Cr, Li, Ni, Sr, V) and toxic (Cd, Pb, Al) elements in different soybean species (*Glycine max*, *Vigna radiata*, *Vigna angularis*, *Vigna mungo*); (ii) to study the existence of significant differences in the content of toxic elements between the different soybean origins (China, Italy, Spain, Australia, Canada, Thailand); and (iii) to assess the toxic risk from the consumption of the studied soybeans considering the intake of the potential toxic elements analyzed. This will guarantee the safe consumption of this new food that is on the rise.

## 2. Materials and Methods

### 2.1. Samples

A total of 90 samples of different soybean species were analyzed. The soybean samples were divided into 13 types of green soybeans, 10 types of yellow soybeans, 4 types of red soybeans, and 3 types of black soybeans. The samples were purchased in different places from Spain and Italy. Table 1 shows the characteristics (origin, cultivation, type of packaging, and place of purchase) of the analyzed samples.

### 2.2. Analytical Procedure

Reagents of analytical-grade quality were used for the treatment of the samples and the analytical procedure.

For each type of soy, 5 g was weighed for each sample and placed in porcelain crucibles (Königliche, Berlin, Germany). Subsequently, they were placed in the stove for 24 h at 70 °C for drying. Then, the samples were subjected to acid digestion with nitric acid (HNO_3_) at 65% (Merck, Darmstadt, Germany) to remove organic matter, until complete acid evaporation has taken place at 100 °C with a heating plate (Nabertherm, Lilienthal, Germany). After digestion, they were introduced into the muffle furnace (Nabertherm, Lilienthal, Germany) set at 420 °C for 48 h (1440 min); in particular, 24 h is needed to reach 450 °C and the temperature is maintained in the remaining 24 h. The goal is to get white ash. Once the desired ashes were obtained, they were diluted with HNO_3_ at 1.5% to reach 25 mL. Each sample was then transferred to a bottle of polypropylene and kept at room temperature until analysis [10,43,44,45,46,47,48].

The determination of trace elements in the proposed soybeans was carried out by ICP-OES (inductively coupled plasma-optical emission spectrometry). The spectrometer used in the trace elements determination was the ICAP 6300 Duo Thermo Scientific (Waltham, MA, USA) with an autosampler CETAX model ASX-520 (Thermo Fisher Scientific, Waltham, MA, USA) equipped with a CID86 chip (Charge Injection Device, Thermo Fisher Scientific, Waltham, MA, USA) with a range of wavelengths from 166 to 847 nm. The following are the wavelengths (nm) of study for each element: Cd (214.4), Pb (220.3), Al (167.0), Cr (267.7), Li (670.7), Ni (221.6), Sr (407.7), Co (228.6), V (292.4), Ba (455.4), and B (249.6).

The ICP-OES instrument was operated with an RF power of 1.2 kW, a gas flow rate of 0.5 L/min, an injection pump speed of 50 rpm, and a 0 s stabilization time. High-purity liquid argon (99.999%) from Air Liquid, Madrid, Spain, was used as the carrier gas.

The analytical procedures used in the determination of elements in soy have been previously validated so as to carry out the analysis. The quality control was based on the recovery percentage obtained after subjecting the reference materials to the same treatment and analysis procedure as the samples. It has been determined that the materials used have the Standard Reference Material (SRM) of the National Institute of Standards and Technology (NIST); specifically, SRM 1515 Apple Leaves, SRM 1567a Wheat Flour, and SRM 1548a Typical Diet were used [10,49]. The standard additions method was used in the quality control in the case of lithium by adding known Li quantities to dehydrated samples of the analyzed legumes. The recovery percentages obtained were over 95% with no significant differences (*p* < 0.05) between the results. This is shown in Table 2.

### 2.3. Statistical Analysis

Statistical analysis was performed using the IBM Statistics SPSS 21.0 program (IBM, Armonk, NY, USA) to study the existence of significative differences in the toxic elements’ contents between the samples (soybean type and origin).

The normality of the data was verified by the Kolmogorov–Smirnov and Shapiro–Wilk test [50]. When there were no normal data, the non-normality of the results was confirmed with a non-parametric study, performed using the Kruskal–Wallis test [51,52]. Since there is no normality, to verify if there are significant differences between groups, a post hoc study using the Mann–Whitney test was conducted [10]. After all of the variables were assessed, the correlation coefficients were determined according to Spearman’s Rho method to determine the extent of the connection between the studied variables and to analyze their behavior against the bilateral distribution.

### 2.4. Dietary Intake Calculations

To evaluate the dietary intake assessment, estimated daily intake (EDI) of each metal was calculated. The last official consumption survey published by the AECOSAN (Spanish Agency for Consumer Affairs, Food Safety and Nutrition) in relation to the Spanish population [53] shows an average consumption of 12.33 g of soy per day in adults. However, given the increase in consumption of this product, we will assume a consumption of 50 g, which is the consumption recommendation made by the AECOSAN for all legumes in the Spanish population [54].

EDI (mg/day) = mean consumption (kg/day) · trace element concentration (mg/kg)

Once EDI has been calculated, the percentage of contribution to the limit intake values for adults of the analyzed metals, set by the main health organizations, were calculated according to the following formula:

Contribution (%) = [EDI (mg/day)/Limit intake value] · 100

Table 3 shows the guideline values of TWI (tolerable weekly intake), BMDL (Benchmark Dose Level), TDI (Tolerable Daily Intake), UL (tolerable upper intake level), RfD (oral reference dose), p-RfD (subchronic and chronic reference dose), and SLI (Safe Level of Intake) set by the EFSA and WHO (World Health Organization) for the studied elements. Most of the intake limit values are expressed per body weight (bw).

## 3. Results and Discussion

### 3.1. Toxic Element Content by Soybean Type

Table 4 shows the trace element mean content (mg/kg wet weight) and standard deviation (SD) of the analyzed soybeans.

Yellow soybeans (*G. max*) recorded the highest Cd (0.03 mg/kg ww) and B (13.5 mg/kg ww) contents. Franzaring et al. (2018) [61] recorded Cd levels in German soybeans of 0.025–1.2 mg/kg, which are higher than the levels found in the present study. However, Franzaring et al. (2018) concluded that the reason behind these Cd levels was the relatively high contamination in Germany due to the mining and metal industry near soybean crops.

Meanwhile, the red soybean (*V. angularis*) is distinguished by its high levels of Co (0.22 mg/kg). Remarkably, they also have the highest values found of Ni (7.21 mg/kg ww), Ba (2.95 mg/kg ww), Li (1.37 mg/kg ww), Cr (0.16 mg/kg ww), and V (0.05 mg/kg ww).

Green soybeans (*V. radiata*) stand out because of their highest Al (7.27 mg/kg ww) content, and black soybeans (*V. mungo*) registered the highest Pb (0.07 mg/kg ww) and Sr (4.56 mg/kg ww) contents.

Otaka et al. (2014) [49], in a study conducted on yellow soybeans from Japan, determined higher concentrations of Al (64.0 mg/Kg dry weight) and Ni (5.02 mg/Kg dw), but similar levels of Sr (4.49 mg/Kg dw). Akinyele and Shokunbi (2015) [62], in a study carried out on legumes in Nigeria, determined in soybeans higher concentrations of Cr (0.58 mg/Kg dw) and Cd (0.02 mg/Kg dw), similar concentrations of Pb (<0.08 mg/Kg dw), and lower concentrations of Ni (0.08 mg/Kg dw), which in turn are much lower than other concentrations detected by Onianwa et al. (2001) [63] in the same country (Cd 0.2 mg/Kg dw and Ni 5.07 mg/Kg dw). Studies in Iran have also shown higher concentrations of Cd (0.11 mg/Kg dw), Ni (12.17 mg/Kg dw), and Pb (0.32 mg/Kg dw) [64].

The statistical analysis shows significative differences (*p* < 0.05) in B and Cd concentrations found in yellow soybeans, which differ significantly from all of the other types analyzed. In green soybeans, significant differences were found in the Cr levels compared to the rest of the legumes, with the lowest content. The Co levels are statistically different among all of the analyzed soybeans. The Ba and Ni concentrations differ statistically between the red soybean and the rest of the analyzed legumes, with the highest contents, and Pb, with the lowest contents. It can be observed that the Sr content of black soybeans differs significantly from all other types. Finally, no significant differences (*p* < 0.05) were found in the contents of Al, Li, and V.

It should be highlighted that all of the species studied are below the parametric values set by European legislation.

### 3.2. Trace Element Content by Origin

Table 5 shows the concentrations (mg/kg of wet weight) and the standard deviations (SDs) of the metals studied according to the origin of the soybeans (China, Italy, Spain, Australia, Canada, Thailand, and unknown origin). Soybean species were excluded from this comparison. As previously noted, all of the soybean varieties investigated are commonly categorized under the umbrella term “soy” and indistinguishably consumed by the public.

By performing statistical analysis of the data between the content of metals of soybeans from different origins, no significant differences (*p* < 0.05) were found in the contents of B, Li, Co, Ni, Pb, and V. Nevertheless, the concentrations of Cr and Al of Italian soybeans differ significantly from all soybeans coming from other countries, and Italian and Canadian soybeans present significant differences in the content of Cd. Finally, the contents of Ba and Sr differ considerably in soybeans from Australia compared to the soybeans of other countries.

Several factors can account for these variations in toxic element levels. Regional disparities such as climate, soil composition, potential industrial activities, and temperature can all impact the concentration of these elements [65,66]. Additionally, we must consider that the different agricultural practices that can be carried out in each country can affect the final composition, especially in those countries outside the European Union. Thus, there may be different practices in the use of pesticides, fertilizers, irrigation techniques, etc., with the potential for the use of poorly controlled and contaminated resources [4]. Finally, we must take into account the possibility that different varieties within each soybean species have been developed in each geographical location, which could lead to a different capacity for metal accumulation.

### 3.3. Dietary Intake Assessment

As described above, the EDI of each metal was calculated using the average consumption of 50 g of soy per day, which is the consumption recommendation made by the AESAN for all legumes in the Spanish population [54]. Furthermore, in accordance with EFSA considerations, for the determination of intake limit values in the adult population, we will consider the standard body weight of 70 kg [67]. Table 6 shows the EDI and percentage contribution of each toxic element for each soybean species.

As a result of the evaluation of the dietary intake of soybeans, assuming that it was the only food consumed within the legume group, the percentage contribution to the intake limit of lithium stands out. Although the amounts of this element are not excessively high, they result in contribution percentages between 31.43–48.57%, with the highest percentage corresponding to red soybeans (*V. angularis*).

The following element that stands out in its contribution to the intake limit is nickel, with contribution percentages between 6.81–39.56%, followed by lead, with contribution percentages between 7.14–10%. The highest contribution percentages correspond, respectively, to red soybeans (*V. angularis*) and black soybeans (*V. mungo*).

Finally, aluminum, with contribution percentages between 2.98–3.6%, and strontium, with contribution percentages between 1.63–2.53%, stand out. The remaining elements have contribution percentages generally below 1%.

Nevertheless, the contribution percentages of each toxic element to the intake limit values do not represent a toxicological risk in any case. Despite this, long-term exposure studies and the evaluation of potential cumulative effects are necessary to determine the food safety of consuming these legumes. This will be especially necessary for those elements with a contribution percentage higher than 10% [68], as is the case with Li and Ni.

## 4. Conclusions

This study provides a comprehensive analysis of potentially toxic elements (Al, B, Ba, Cd, Co, Cr, Li, Ni, Pb, Sr, V) in different soybean species (*Glycine max*, *Vigna radiata*, *Vigna angularis*, *Vigna mungo*) sourced from six countries. Barium and aluminum were generally found at the highest concentrations, with notable differences across countries of origin. These results highlight the influence of environmental and agricultural factors on elemental accumulation in soybeans. Although typical soybean consumption does not appear to pose immediate health risks, the presence of elements like lithium suggests that increased consumption of soy-based foods could elevate exposure to potentially harmful levels. This underscores the need for continued monitoring of toxic elements in soybeans and similar crops, and toxicological studies on the long-term consumption of soybeans containing these levels. Future efforts should focus on implementing stricter agricultural and environmental management practices to limit contamination of crops from industrial and environmental sources. Additionally, further research into safe thresholds for these elements in food products, along with regular updates to regulatory standards, will help mitigate potential health risks associated with long-term exposure to non-essential and toxic elements in soybeans.

## Figures and Tables

**Table 1:** Characteristics of the analyzed samples.

Type of Soybean	Sample Code	Number of Samples	Origin	Cultivation	Type of Packaging	Place of Purchase
Yellow	O1	3	Italy	Organic	Plastic	Hypermarkets
	O2	3	Italy	Organic	Plastic	Organic supermarkets
	O3	3	Italy	Organic	Plastic	Organic supermarkets
	C1	3	Canada	Conventional	Plastic	Hypermarkets
	O4	3	Spain	Organic	Plastic	Hypermarkets
	C2	3	China	Conventional	Plastic	Chinese supermarket
	O5	3	Spain	Organic	Plastic	Herbalist store
	C3	3	Unknown	Conventional	Bulk	Chinese supermarket
	C4	3	China	Conventional	Plastic	Chinese supermarket
	C5	3	Unknown	Conventional	Bulk	Popular markets
Green	O6	3	Spain	Organic	Plastic	Herbalist store
	C6	3	Australia	Conventional	Plastic	Hypermarkets
	O7	3	Spain	Organic	Plastic	Herbalist store
	O8	3	China	Organic	Plastic	Organic supermarkets
	C7	3	Unknown	Conventional	Bulk	Chinese supermarket
	O9	3	Spain	Organic	Plastic	Herbalist store
	C8	3	Unknown	Conventional	Bulk	Chinese supermarket
	C9	3	Thailand	Conventional	Plastic	Chinese supermarket
	C10	3	China	Conventional	Plastic	Chinese supermarket
	C11	3	Unknown	Conventional	Bulk	Popular markets
	C12	3	Unknown	Conventional	Bulk	Popular markets
	O10	3	Spain	Organic	Plastic	Hypermarkets
	O11	3	Spain	Organic	Plastic	Hypermarkets
Black	C13	3	Thailand	Conventional	Plastic	Chinese supermarket
	C14	3	China	Conventional	Plastic	Chinese supermarket
	O12	3	Spain	Organic	Plastic	Popular markets
Red	C15	3	China	Conventional	Plastic	Chinese supermarket
	C16	3	Unknown	Conventional	Bulk	Popular markets
	C17	3	Unknown	Conventional	Bulk	Popular markets
	C18	3	China	Conventional	Plastic	Chinese supermarket

**Table 2:** Recovery (R) percentage study of the reference materials used.

Metal	Material	Concentration Found (mg/kg)	Certified Concentration (mg/kg)	R (%)
Al	SRM 1515 Apple Leaves	286 ± 9	285.1 ± 26	99.7
B		27.0 ± 2.0	27.0 ± 1.5	99.9
Sr		25.0 ± 2.0	24.6 ± 4.0	98.3
Ba	SRM 1548a Typical Diet	1.10 ± 0.10	1.13 ± 0.09	102.5
Ni		0.37 ± 0.02	0.38 ± 0.04	102.3
Pb		0.044 ± 0.000	0.044 ± 0.013	98.9
Cd	SRM 1567a Wheat Flour	0.026 ± 0.002	0.026 ± 0.008	98.4
Co		0.006 ± 0.00	0.006 ± 0.002	102.4
V		0.011 ± 0.00	0.011 ± 0.00	99.4
Li	Standard Addition Method	0.2 ± 0.02	0.19 ± 0.03	95.0

The limits of quantification (LOQ) (mg/L) were as follows: Al (0.012), B (0.012), Ba (0.005), Cd (0.001), Co (0.002), Cr (0.008), Li (0.013), Ni (0.003), Pb (0.001), Sr (0.003), and V (0.005).

**Table 3:** Guideline values of the studied potential toxic elements.

Metal	Parameter	Intake Limit Value	Organization	Reference
Cd	TWI	2.5 μg/kg bw/week	EFSA	[55]
Pb	BMDL	0.50 μg/kg bw/day ^1^ 0.63 μg/kg bw/day ^2^ 1.50 μg/kg bw/day ^3^	EFSA	[56]
Al	TWI	1 mg/kg bw/week	EFSA	[57]
Cr	TDI	0.3 mg/kg bw/day	EFSA	[58]
Li	p-RfD	2 μg/kg bw/day	EFSA	[37]
Ni	TDI	13 μg/kg bw/day	EFSA	[42]
Sr	TDI	0.13 mg/kg bw/day	WHO	[40]
B	UL	10 mg/day	EFSA	[59]
V	RfD	7 μg/kg bw/day	EFSA	[37]
Ba	TDI	0.2 mg/kg bw/day	EFSA	[37]
Co	SLI ^4^	600 μg/day ^4^	EFSA	[60]

^1^ For neurotoxicity; ^2^ for nephrotoxicity; ^3^ for cardiovascular effects; ^4^ although an official parameter has not been established, it has been estimated by EFSA as a safe acceptable daily oral intake, with a minimal level of risk.

**Table 4:** Mean concentration (mg/kg) in the analyzed samples by type.

Element	Soybean Types			
	Green (*V. radiata*)	Yellow (*G. max*)	Red (*V. angularis*)	Black (*V. mungo*)
Cr	0.09 ± 0.02	0.15 ± 0.09	0.16 ± 0.02	0.14 ± 0.04
Co	0.03 ± 0.007	0.05 ± 0.02	0.22 ± 0.10	0.09 ± 0.01
Al	7.27 ± 4.51	6.31 ± 2.60	5.96 ± 0.99	6.55 ± 1.22
Cd	0.0003 ± 0.001	0.03 ± 0.01	0.005 ± 0.00003	0.001 ± 0.002
Pb	0.06 ± 0.02	0.06 ± 0.01	0.05 ± 0.005	0.07 ± 0.009
B	7.74 ± 2.12	13.5 ± 4.06	6.88 ± 0.93	7.62 ± 0.46
Ba	1.66 ± 0.74	1.82 ± 0.34	2.95 ± 0.74	1.72 ± 0.50
Li	1.23 ± 0.82	1.05 ± 0.88	1.37 ± 0.88	0.88 ± 0.58
Ni	1.24 ± 0.35	1.88 ± 1.43	7.21 ± 1.57	2.10 ± 0.98
Sr	3.23 ± 1.62	3.42 ± 1.92	3.04 ± 0.48	4.56 ± 1.03
V	0.04 ± 0.03	0.05 ± 0.04	0.05 ± 0.04	0.04 ± 0.04

**Table 5:** The concentrations (mg/kg wet weight) and the standard deviations (SDs) of the metals studied according to the origin of the soybeans across all species.

	China	Italy	Spain	Australia	Canada	Unknown	Thailand
Cr	0.11 ± 0.03	0.25 ± 0.13	0.11 ± 0.02	0.07 ± 0.005	0.11 ± 0.02	0.12 ± 0.038	0.13 ± 0.066
Co	0.07 ± 0.06	0.05 ± 0.006	0.05 ± 0.03	0.01 ± 0.00	0.04 ± 0.005	0.1 ± 0.126	0.061 ± 0.041
Al	5.93 ± 2.07	8.59 ± 2.68	7.49 ± 4.73	7.46 ± 0.44	5.94 ± 2.51	6.32 ± 3.33	4.96 ± 1.02
Cd	0.02 ± 0.02	0.03 ± 0.004	0.006 ± 0.01	<LOD	0.03 ± 0.0005	0.008 ± 0.0127	<LOD
Pb	0.06 ± 0.01	0.07 ± 0.01	0.06 ± 0.02	0.06 ± 0.005	0.06 ± 0.01	0.061 ± 0.014	0.058 ± 0.0098
B	10.2 ± 4.42	8.79 ± 4.39	9.07 ± 3.74	9.52 ± 0.38	15.9 ± 1.09	9.41 ± 3.84	7.57 ± 0.48
Ba	1.98 ± 0.68	1.82 ± 0.41	1.59 ± 0.66	3.23 ± 0.22	1.47 ± 0.27	2.01 ± 0.89	1.88 ± 0.32
Li	0.78 ± 0.63	1.42 ± 0.71	0.93 ± 0.83	1.32 ± 0.53	1.15 ± 2.00	1.45 ± 0.64	1.69 ± 1.11
Ni	2.63 ± 2.15	2.03 ± 0.97	1.77 ± 1.47	1.62 ± 0.10	1.34 ± 0.12	2.95 ± 3.38	2.44 ± 0.45
Sr	2.89 ± 1.54	3.37 ± 0.37	3.89 ± 1.76	<LOD	4.64 ± 0.74	3.5 ± 1.54	3.9 ± 0.97
V	0.05 ± 0.03	0.06 ± 0.06	0.03 ± 0.03	0.01 ± 0.02	0.04 ± 0.04	0.049 ± 0.029	0.029 ± 0.033

**Table 6:** The percentage of contribution and EDI (estimated daily intake) of each trace element given the guideline values for each soybean species.

Element	Guideline Values	Soybean Types							
		Green (*V. radiata*)		Yellow (*G. max*)		Red (*V. angularis*)		Black (*V. mungo*)	
		EDI (mg/day)	% Contribution	EDI (mg/day)	% Contribution	EDI (mg/day)	% Contribution	EDI (mg/day)	% Contribution
Cr	21 mg/day	0.0045	0.021	0.0075	0.036	0.008	0.038	0.007	0.033
Co	0.6 mg/day	0.0015	0.25	0.0025	0.42	0.011	1.83	0.0045	0.75
Al	70 mg/week	2.52 *	3.6	2.21 *	3.16	2.09 *	2.98	2.29 *	3.27
Cd	0.18 mg/week	0.0001 *	0.056	0.01 *	5.56	0.0018 *	1	0.00035 *	0.19
Pb	0.035 mg/day	0.003	8.57	0.003	8.57	0.0025	7.14	0.0035	10
B	700 mg/day	0.39	0.056	0.68	0.097	0.34	0.048	0.38	0.054
Ba	14 mg/day	0.083	0.59	0.091	0.65	0.15	1.07	0.086	0.61
Li	0.14 mg/day	0.062	44.28	0.052	37.14	0.068	48.57	0.044	31.43
Ni	0.91 mg/day	0.062	6.81	0.094	10.33	0.36	39.56	0.1	10.99
Sr	9.1 mg/day	0.16	1.76	0.17	1.87	0.15	1.65	0.23	2.53
V	0.49 mg/day	0.002	0.41	0.0025	0.51	0.0025	0.51	0.002	0.41

* In these cases, the value indicated corresponds to the estimated weekly intake (EWI).

## Data Availability

The original contributions presented in this study are included in the article. Further inquiries can be directed to the corresponding authors.
